# Community Care for People with Complex Care Needs: Bridging the Gap between Health and Social Care

**DOI:** 10.5334/ijic.2944

**Published:** 2017-07-21

**Authors:** Kerry Kuluski, Julia W. Ho, Parminder Kaur Hans, Michelle LA Nelson

**Affiliations:** 1 Bridgepoint Collaboratory for Research and Innovation, Lunenfeld-Tanenbaum Research Institute, Sinai Health System, CA; 2 Institute of Health Policy, Management and Evaluation, Dalla Lana School of Public Health, University of Toronto, CA; 3 Daphne Cockwell School of Nursing, Ryerson University, CA

**Keywords:** multimorbidity, homecare, transitions, social care, social determinants of health, integrated care

## Abstract

**Introduction:**

A growing number of people are living with complex care needs characterized by multimorbidity, mental health challenges and social deprivation. Required is the integration of health and social care, beyond traditional health care services to address social determinants. This study investigates key care components to support complex patients and their families in the community.

**Methods:**

Expert panel focus groups with 24 care providers, working in health and social care sectors across Toronto, Ontario, Canada were conducted. Patient vignettes illustrating significant health and social care needs were presented to participants. The vignettes prompted discussions on i) how best to meet complex care needs in the community and ii) the barriers to delivering care to this population.

**Results:**

Categories to support care needs of complex patients and their families included i) relationships as the foundation for care, ii) desired processes and structures of care, and iii) barriers and workarounds for desired care.

**Discussion and Conclusions:**

Meeting the needs of the population who require health and social care requires time to develop authentic relationships, broadening the membership of the care team, communicating across sectors, co-locating health and social care, and addressing the barriers that prevent providers from engaging in these required practices.

## Introduction

Millions of people worldwide have complex care needs [1,2,3] resulting from multiple concurrent chronic conditions, functional and cognitive impairments, mental health challenges and social vulnerability [4]. Illness has a significant impact on the lives of individuals, over and above managing treatments and medicines [5] including social participation, relationships and societal contributions [6]. Despite the growing numbers of people who present with complex health and social care needs, health systems continue to deliver care that predominantly focuses on one illness at a time or prioritizes medically oriented care (management of disease and symptoms) over socially oriented care (attention to quality of life and social support).

There is widespread consensus that improving care for people with complex care needs requires integration of health and social care services [7,8]. The need for such integration becomes more apparent at particular points of a person’s care journey, especially as they transition from one care site to another. For example, when preparing for hospital discharge, the mobilization of both health care services (such as nursing or home physiotherapy) and social care services (such as assistance with instrumental activities of daily living or making adaptations to the home environment) may be required to support ongoing care needs. Failing to mobilize health and social care in the community may result in a hospital discharge delay [9,10,11,12,13,14], and once discharged home could result in hospital readmission [15,16].

Mobilizing health and social care in the community has proved challenging in the study context (Toronto, Ontario, Canada) as well as other jurisdictions worldwide, partly because homecare services tend to be medically oriented. Supports for nursing care, physical rehabilitation and *activities of daily living* (bathing, toileting and personal hygiene), are more likely to be publicly funded entitlements for those who meet specified eligibility criteria. On the other hand, *instrumental activities of daily living* (IADLs) such as: meal preparation, transportation, paying bills, partaking in social activities, and home maintenance, are only partially (or not) subsidized by the government. Moreover, some patients find services to be inaccessible due to factors such as cost or location. Receiving care may be contingent on the presence and capacity of family and friends to connect them to services or provide it directly. To further complicate matters, patients may find themselves needing support for public programs that lie *outside* of health care entirely, including workplace reintegration, obtaining or maintaining adequate and affordable housing, making home adaptations, and seeking financial support. While these types of services are situated *outside* of health care they are inexplicitly tied to one’s ability to maintain overall health. Since there is no standard definition for “social care” we define it in this paper as services outside health care as well as services to support IADLs. Importantly, the absence of needed social care has been linked to increased use of inappropriate medical care, which is both unnecessary and typically more expensive [17].

There remains a poor understanding of what optimal care in the community entails for people that present with significant health *and* social care needs, particularly when needs span beyond what the health care system typically provides. As a start, it is important to garner the perspectives of experienced health and social care providers, who work with this population, to gain insight into what community supports are needed and what gets in the way of providing them.

In this study we presented health and social care providers with composite patient vignettes characterized by significant health and social care needs, nearing hospital discharge. The vignettes were used to guide a discussion on i) how best to meet the needs of people with complex care needs in the community and ii) the barriers experienced when trying to deliver care to this population.

## Methods

### Design

This study is the second phase of a two-phased sequential mixed methods design [18]. In the first phase a series of patient vignettes (composite descriptions), based on previously conducted patient interviews [19] were created. Vignettes have been used previously in health services research to outline patient characteristics and prompt discussion around decision making and care planning with expert participants [20]. The vignettes featured a range of complex patient cases across young adult (18 – 44 years of age), mid-life (45 – 64 years of age) and older adult age groups (65 years of age and above), with a variety of health and social challenges. As noted above, the creation of the vignettes was informed by a previous study led by the lead author. In this original analysis patient experience appeared to vary by two key factors: illness trajectory (sudden illness or ongoing illness) and life circumstances (related to age/stage of life, social, financial, practical, and other non-bio-medical circumstances). To create the vignettes, the full set of interviews was categorized by illness trajectory and age group (young, mid-life and older) and a sub-set of interviews were selected from each category for additional in-depth analysis. Patient vignettes were created to reflect the patterns that emerged from this analysis as opposed to individual cases (so that confidentiality of patients could be protected). These vignettes were subsequently reviewed and approved for content by health and social care providers at Bridgepoint Active Healthcare (an intermediate care facility providing complex continuing care and rehabilitation located in Toronto, Canada) in preparation for this current study. The full vignettes are available as an appendix and a summary can be viewed in Table 1. Further details of how the vignettes were designed are outlined in a report that was published online [21]. The current study entailed two in-depth expert panel sessions (similar to the structure of a focus group).

**Table 1:** Vignette Characteristics.

Fictional Name	Age	Main Medical Characteristics	Main Social Characteristics	Discharge Location Needs

Maggie	29 years	Intellectual and physical disability	Poverty, caregiver stress	Environment close to her peers
Aaron	33 years	Infection, addictions	Homelessness, no family	Stable home
Mandeep	40 years	Traumatic brain injury	Behavioral issues, caregiver stress	Home that is safe for children and wife
Cynthia	45 years	Stroke, aphasia	Financial strain, dependents	Accessible/adapted home
Kate	55 years	Diabetes, obesity, depression	Bed bugs, hoarding, job loss, children taken away	Accessible/adapted home
Grace	70 years	Multiple Sclerosis	Caregiver stress, caregiver health decline, isolated (rural)	Accessible/adapted home, stay with husband
Min Yee	80 years	Hip fracture, arthritis, osteoporosis, diabetes, hypertension	Sibling disagreement, deferred decision making	Culturally appropriate long-term care

### Sampling and Recruitment

An environmental scan of organizations that deliver health and social care in the community in the Greater Toronto Area of Ontario was conducted to identify the sampling frame. Searches for organizations were conducted from January to February 2016 using internet sources including the Community Navigation and Access Program (www.4seniors.org), Canadian Research Network for Care in the Community (http://www.ryerson.ca/crncc/), and Toronto Central Health Line (www.torontocentralhealthline.ca). In addition to an environmental scan, a snowball sampling strategy was conducted, that consisted of asking the Professional Practice Leader for social workers at the aforementioned intermediate care facility that serves a complex patient population, to identify providers who work as care coordinators or direct care providers for people with complex care needs. The project manager for a hospital discharge program at the same site was also asked to provide a list of contacts. The environmental scan and snowball sampling strategy resulted in a list of providers from a variety of settings including: acute and rehabilitation hospitals, homecare, housing with care (supportive housing/assisted living), primary care, mental health, other community support services (such as meals and transportation), as well as mental health, legal and workplace integration services.

Email invitations from the research coordinator detailing the study purpose were sent to 71 providers who were given two options (meeting dates) to participate. 24 responded and agreed to participate, 13 declined and 34 did not respond to either the initial or follow-up invitation.

This study received ethics approval from the Mount Sinai Hospital Research Ethics Board. All ethical requirements were adhered to throughout the study and consent was received from participants prior to the initiation of study activities.

### Data Collection

Each expert panel session was approximately 3 hours in length. Participants received printouts of the 7 case vignettes which were used as prompts to guide the discussion on the resources required to adequately support patients with complex care needs in the community following hospital discharge, as well as related barriers.

The sessions were audio-recorded and led by the lead author (KK), a trained and experienced qualitative researcher who has facilitated expert panel sessions and focus groups with care providers in the past. Two members of the research team (JH and PH) took extensive notes during the session as a supplement to the audio-recordings.

### Data Analysis

Qualitative content analysis activities were implemented. The audio-recordings were transcribed verbatim. The lead author removed identifying information and checked the transcripts for accuracy against the original audio-recordings. To ensure trustworthiness of the data, three members of the research team (KK, JH and PH) reviewed the transcripts independently and made note of key categories (e.g., data derived codes). The team members coded the transcripts inductively, in order to capture the range of categories identified by the participants [22]. Following independent coding, the team met to discuss, map out, and compare categories that emerged across the two focus groups and iron out discrepancies. A consolidated codebook was created and verified by each team member. Next, the lead author coded both transcripts using NVivo 11 software to organize the findings. After both transcripts were coded using this codebook, the lead author compared, aggregated, refined, and organized the codes into higher-level descriptive categories.

## Results

A total of 24 health and social care providers participated in one of the two expert panel sessions (10 and 14 participants in the sessions respectively) that took place in February 2016. The large number of participants in each group allowed for greater variation in participant characteristics, supporting maximal variation of participant perspectives. Table 2 provides a summary of participant characteristics.

**Table 2:** Participant Characteristics.


**Sex** *n = 24*	Male = 2 Female = 22
**Age** *n = 23*	Mean = 47.78 Median = 51 Std Dev = 12.4 Range = 22 – 64 years
**Profession** *n = 21*	Nursing = 5 Social Work = 8 Other^1^ = 8
**Years working as a Health or Social Care Provider** *n = 23*	Mean = 18.2 years Median = 15 Std Dev = 12.26 Range = 2 – 40 years
**Sector** *n = 23*	Community Services^2^ = 10 Hospital^3^ = 6 Other^4^ = 7
**Role** *n = 24*	Director = 5 Educator/Resource Specialist = 5 Program Facilitator/Leader = 4 Care Coordinator and Transitions Support = 5 Direct Service Provider = 5


^1^ includes occupational and recreational therapy, law, education, and midwifery. ^2^ includes some combination of housing, daily living and social supports. ^3^ includes acute care and post-acute/rehabilitation. ^4^ includes homecare, legal services, mental health and addictions, aboriginal health.

**Figure 1:**
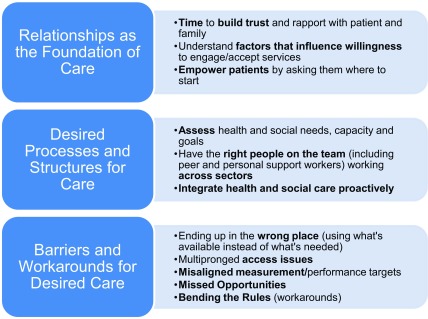
Key Categories Identified by Health and Social Care Providers in the Care of People with Complex Care Needs.

The three broad categories of results with corresponding elements that were determined from both expert panel sessions and across multiple composite vignettes included: i) relationships as the foundation for care, ii) desired processes and structures of care, and iii) barriers and workarounds for desired care. These categories are outlined in Figure 1. We describe the corresponding components of each of these categories below with illustrative quotes. While the illustrative quotes were often pulled in response to specific vignettes, the categories surfaced across all vignettes, and thus appear to represent general fundamental components of care for complex patient populations.

## Category 1: Relationships as the Foundation for Care

Integral to the care of people with complex care needs was the establishment of a strong therapeutic relationship. The development of trust between care providers and care recipients was essential, but required time which was often in short supply. One of the participants noted that a strong relationship with the client could serve as an entree to their receptivity to services.

“So it would be good to have a person that you can spend time building that rapport. So even if he [client] just opens the door a crack and that person has that in, and then you start off with a 5, 10 minute conversation, and then you get the person to agree to meet you again. And maybe the next time you meet, it’s a 20 minute conversation. And you build that trust. And you’re able to take that time.”

Participants stressed that relationships needed to be built with the patient’s family/support network as well:

“…we actually have multiple family meetings. And it’s like a step by step process. So if we can’t get all this done in one meeting, we actually have to have multiple [meetings]. And this situation has probably happened to us a couple of times. And we give opportunities to the family to say…what they want to say. But they may not say that at the very beginning. But the longer the patient stays in hospital, a lot of things start to unravel. Like the family dynamics.”

Being aware of the factors that shape the willingness of patients and their families to engage, discuss, or accept services, was noted. For example, cultural expectations and norms were integral to understanding client behaviors and openness to receiving care:

“You’ve got to deal with the fact that there’s a cultural thing at play here. How people perceive disability or mental health reactions in different cultures. I mean they may not want to tell you what’s going on.”

Resistance to care was also a product of a variety of other factors including; previously poor care experiences, lack of provider care continuity, as well as significant changes to a person’s care plan. Being open to significant changes in care settings (such as moving from home to a long-term care facility) required (as one provider put it): “*planting that seed*.”

Finally, empowering people by providing them with some degree of choice in the types of services provided and locations of care was emphasized. Since the patients featured in our study had to deal with a myriad of health and social challenges, providers suggested asking clients where to start:

“I think “Kate” needs to have some control. Because all these losses are just, you know, getting accumulated on her. And I like the idea of asking her even what do you want to tackle?”

## Category 2: Desired Processes and Structures of Care

This category included the tangible components of care including: a comprehensive assessment of the needs and decision-making capacity of patients, optimization of the structure, members and roles of the care team, support for formal and informal caregivers, and an appropriate continuum of services delivered proactively.

### Assessing Need and Capacity

In order to fully understand the breadth of need and capacity of patients, assessments of physical needs and symptoms, mental health status, social context, and factors that influence behaviors and receptivity to care (culture, personal goals, and expectations) was recommended:

“I agree though that when you’re starting with the client, if you begin there, you should be doing a proper psychosocial and medical assessment. So that doesn’t just include the physical demands, the mental demands, but where are their passions, where’s the spark in their life, what are the things they like to do, what gives them meaning? All those things need to be included in the picture right from the beginning. Because they all work out and they improve quality of life.”

Having the appropriate approaches and processes in place to assess the person’s ability to make decisions about their care was important. Capacity was described as fluctuating, and best assessed by a provider who knew the patient well enough to understand the nature of their illness and its nuances. Consent and capacity was generally poorly managed and not always determined by the right care provider(s):

“And that’s the problem that’s come up more and more as of lately because it used to be that like the team and the physician would make the decision if they felt someone was capable. So if you’re working with someone for 4 to 6 weeks, and you see them day in and day out… And people, sometimes their cognition fluctuates or they have better times of the day. You kind of really get to know someone. You know, their safety judgement and things like that. But now instead of making the decision if they’re capable, now the [Homecare Agency] comes in, have never met this patient until that capacity meeting, and they’re like, “Oh, they’re capable.” We’re like ahh! Like probably not very safe. And it’s a decision that’s been taken away and the [Homecare Agency] has taken over that component.”

Incorrectly determining capacity could have repercussions for the patient (who may overestimate their ability to manage their care needs), as well as the family (if available) who may be left with significant care responsibilities.

Regardless of the capacity or decision making preference expressed by the patient, providers felt that it was important for the patient to be “at the table” when decisions were being made. At times, regardless of capacity, patients deferred decisions to family. When sibling conflict arose over care choices and settings, regular family meetings and discussions with the patient present was suggested as a means to help ameliorate these misunderstandings.

Giving the strain that is felt by both formal and informal care providers, the participants emphasized the need to support and enhance the capacity of both parties.

“I’d like to see some funding around building the capacity of caregivers, both informal and formal caregivers. And I think it was to [Participant’s] point as well about the formal caregivers. So I’d like to see funding too that goes to associations or to organizations, agencies that put in place training, specialized training for care providers to prevent burnout around specialized high risk, high needs, complex patients, and how best to empower those caregivers.”

### Having the Right People on the Team and Working across Sectors

Participants emphasized that “*comprehensive health teams with the nurse, the doctor, the social worker, the navigator,”* could be enriched by including persons who were typically overlooked such as Personal Support Workers (unregulated homecare providers in the study context) and the inclusion of *peer* support workers described as:

“…a person with lived experience who is available in [an] emergency. So that a person experiencing this trauma in their life, a mental health challenge, and they’re all discombobulated, they talk to another person – I’ve been where you’re at, guy. And it calms them down … like you can’t bullshit me because … I’ve been through it too.”

To ensure that the *“the wheels don’t fall off,”* participants noted the team needed to coordinate care across the various sectors (hospital, community, etc.) used by the patient.

“I think it can work in the community if it begins in the hospital. We have the discussions there with the family, and then we move into the community. We meet in the community with the person, the family, and sort of juggle our basket of services. So this person is getting services that help them remain home.”

A key challenge related to care coordination was being unsure about who on the team was doing what:

“You know, it’s all right to have …different people working with someone but it needs to be coordinated, you know, and we have to know what we’re doing with each other.”

As part of a coordinated care strategy, participants suggested that ‘interim care options’ such as housing with services could ease the burden after a hospital transition, and enable patients to experience a more gradual shift from a fully supported formal care environment to self-care at home with largely informal supports. It would also potentially ease the burden on the family, whose lives were frequently impacted. An interim option could also ease an eventual shift to a higher level of care (such as care in a long-term care facility), as is the case illustrated here:

“Gradually she could transition to a place that was familiar to her without making that immediate cut from the home environment. So you know, if you could say yes, we will discharge you home but here, you know, 3 days a week, you’re going to go to this program while mom works. You’re going to get to know the team, and gradually increase as her dependence drops. And that would be a very nice transition as opposed to the current state, which is sort of wherever there’s a bed, off you go.”

### Integrating Health and Social Care Proactively

Early and proactive planning of care was emphasized. Participants noted that proactive care could occur earlier in the disease trajectory when a patient was first flagged by a health professional or earlier in the episode of care (such as the early part of hospitalization). Further to that, identification of people who may be in need of care could potentially occur in a more natural setting such as a school, library and other areas of social activity and would support a population health approach to care and risk mitigation. More specifically, proactive care could potentially prevent *unnecessary* decline or, on the other hand, help prepare people for *inevitable* decline:

“…looking at the fact that it is a degenerative neurological condition. She may have improved slightly but her ill [illness trajectory] is down. And this will have huge impacts. So it might be that temporarily she will be a little better at home. The reality is she will deteriorate. Who’s monitoring that level of deterioration and what is the point at which she needs long term care?”

Finally, participants emphasized the utility of col-locating health and social care services. The participants reflected on existing programs that were structured this way and worked well. These included primary care practices with legal services embedded within or housing models (apartment buildings) with care services on site. Participants remarked that although these types of socially oriented and co-located programs were ideal, they had limited capacity, strict eligibility requirements, and lengthy wait times.

## Category 3: Barriers to and Workarounds for Desired Care

Participants described perverse practices, policies, and rules that limited the extent to which therapeutic relationships could be developed, services accessed and integrated, and client needs met. These barriers were experienced in every day practice and limited provider’s aspirations for integrated health and social care detailed in the aforementioned categories.

### In the Wrong Place

A lack of appropriate services/settings often resulted in patients using what was *available* as opposed to what was *truly needed* or appropriate:

“The waiting lists are ridiculous. So I mean the waiting lists are 5, 10 years. And then, you know, she’s almost 40. So she’s in a different demographic and a different set of issues with aging. But I think if we could build up the network of group homes, it would help our long term care system, our tertiary system.”

Participants shared examples of sub-optimal post-hospital care settings fueled by increasingly stringent hospital length of stay targets:

“We’re dealing with a hospital right now that’s discharging to a retirement home that was de-licensed. How can they possibly think that that’s an appropriate place for anyone when the place has lost its license?”

### Multipronged Access Issues

Multiple factors impacted access to services. Eligibility criteria limited access by: disease type (e.g., services for acquired brain injury only), symptoms (e.g., physically but not cognitively impaired), age (65 years and over), and financial status (below a certain income threshold). As noted by one provider, how financial status was assessed was particularly problematic for people who experienced a sudden health event or injury:

“…sometimes when applying for any kind of funding, what we see is they want the person’s financial statement from the year before. So Mandeep [fictional vignette name] might have made a great amount of money as a contractor. And so his past income tax might make him ineligible for certain programs. Whereas the current financial state is much, much different.”

Access also varied geographically as large urban centers had more community care options than adjacent communities or smaller rural areas. Geographic variation in available care (shaped by different sets of programs, providers, and eligibility criteria) was illuminated when patients were discharged from one region to another:

“Sometimes people are going home 3, 4, 5 days without appropriate supports because the hospital has a mandate to get you out, right. And so in the meantime, in that period of time, it takes 3, 4, 5, longer sometimes, right, for them to get hooked up in the right geographical area. And that’s another thing I think, is that each [homecare agency] has… Like one will give you 7 hours a week, one will give you 2 hours a week. So there’s such a variety depending on where you live, what you have the right to in terms of services.”

Participants shared unfair and discriminatory practices, particularly toward people with mental health and addictions issues. These patients, were at times, denied access to services such as assisted living or supportive housing (housing with care supports), given concerns among landlords of unpredictable behaviors and unpaid rents. Participants felt that there were few regulations in place to prevent or address such occurrences. When patients lived in poor living conditions (e.g., characterized by hoarding and bed bugs), providers could opt out of providing a home service because of workplace health and safety concerns.

Finally, participants noted that patients and providers (including themselves) did not know the full complement of services available or how to access them. Having an up-to-date, reliable repository of information (such as a website) with clear descriptions of services, guidelines on how to access them, and their eligibility requirements was recommended.

### Misaligned Measurement

Providers were expected to meet certain performance targets as well as measure peoples care needs in particular ways. In the example detailed here, the provider indicates that certain symptoms such as incontinence elevate a person’s complexity score according to a particular tool used in practice, but can be misleading as other factors may be more informative of someone’s needs:

“And if someone automatically scores a [type of score] because they have incontinence issues, that’s nothing. That’s what we do, right. It doesn’t make you a high needs person because you have incontinence issues. So the tool itself, we don’t even think fits for most of our consumers. But that’s what we’re being forced to do.”

Providing service for certain populations was challenging (e.g., family caregiver support) due to narrowly defined service codes that were linked to provider/organization reimbursement.

“The [regional health authority] funding requirements are so tight. So you know, we have these service codes that you have to fall within, right. So I know in our sector, we don’t have any service code for caregiver support. Right? But so much of what we need to do is caregiver support. But we have no way to capture that. We have no way. And then we have to meet these service targets in order to continue to get the funding. So you have to be really creative with how you’re delivering those services so that you’re capturing and responding to the needs that you need to respond to, and still meet those [regional health authority] targets.”

### Missed Opportunities

Providers reflected on the reactive nature of the health care system and identified missed opportunities for early intervention. Missing early opportunities to intervene had a domino effect:

“I think that this is a very typical patient. And years earlier, at one point she was diagnosed with diabetes. So that was a door of opportunity at that point. And she should have had a good care plan, assessment, evaluation at that point. Because some people manage diabetes and they’re Mary Tyler Moore, right. You know, their life is wonderful. But others, they struggle. And the depression that goes with diabetes, and the management, and the doctor’s appointments, and the kidney functions and the eye exams. It just falls apart fast. And if someone is not well managing themselves anyway, it comes to this.”

### Bending the Rules

Finally, working outside structural constraints by “bending the rules” or taking an extra step was required to make services work better for clients:

“So if you look at each individual as what does this individual need to be successful? And it’s sometimes so unique. And then we have to maybe bend a bit, be flexible rather than being very rigid and working in silos. Because then it does not stretch enough. But at the same time, for that small thing, you can’t, you know, find somebody to come step in and do it. Because these services which are already involved, they need to take one extra step to make it a success. Because they don’t do that one extra step, the person is again put back into the system to go back to the ER and all those kinds of things.”

## Discussion

This study brought together a range of experienced care providers to examine and discuss the ‘fundamentals’ of community-based care for people with complex health and social care needs. The discussion itself and the categories of data that evolved provide a framework for the design of interventions and programs for people with complex care needs. Additionally, this study highlights the myriad of “workarounds” and the lengths that health and social care providers go to in their work with patients and families.

## The Relationship

Participants spent a considerable part of the discussion emphasizing *how* care should be delivered. They emphasized the need for strong therapeutic relationships supported by consistent care providers and time; necessary elements to develop trust and openness to care options. These insights are consistent with frameworks of person and family centered care; frameworks which have gained increasing attention as a core domain of care quality since the release of the Institute of Medicine’s seminal report [23]. A growing body of research on persons with complex care needs sheds light on continued challenges in achieving person centeredness in practice including poor communication with providers and low continuity of care [24]. Our study adds important insights into the concept of person centeredness, particularly the types of things that need attention when engaging patients and families. Determining patient capacity to make decisions, working with families to resolve conflicts and make care decisions, as well as unpacking a person’s culture, social role, and previous experiences is required to understand factors that shape peoples willingness to engage in care planning. Empowering patients through choice of service, location of care, unpacking personal goals and priorities, and allowing time for this process was strongly encouraged; but represents a departure from our current medical care system, which prioritizes short episodes of care delivery, provider driven care decisions, and rewards efficiency and cost-effectiveness in service utilization.

## The Processes and Care Components

Participants outlined a number of processes and approaches that would enable better care for people with complex care needs. As a basis to care, a comprehensive assessment was recommended. While many tools to assess patients are utilized across different programs and settings we reflect on one example due to its widespread international use; the internationally suite of Resident Assessment Instruments (RAI) developed by interRAI research collaborative. These tools are used in a variety of health settings across the world, including the study context, to assess the bio-psycho social characteristics of clients. [25] A Canadian study by Kontos [26] noted that the RAI tools do not account for patients personal preferences or the important insights of Personal Support Workers who interact regularly with them, leading to implications for care quality. Furthermore, the more nuanced aspects of client needs and characteristics (including culture, expectations, goals, etc.) are missing in a formalized manner from standard care assessments such as the RAI. As noted by Turcotte et al [27] properly assessing *and* addressing health and social needs is necessary to meet the ongoing care needs of this population. A recent synthesis of European evidence on care for patients with complex care needs [8], noted that an appropriate assessment of risk inclusive of non-medical factors is required. Ignoring social risk factors have real implications, as noted by the participants in our study, who indicated that overlooking social needs may culminate into a missed opportunity to mitigate a medical crisis later on.

Beyond assessments, members of the care team need to be appropriately supported and equipped to work effectively with persons with complex care needs and their caregivers. For instance Woo et al [28] in their study on older adults and service providers in Hong Kong, identified a need for educational opportunities for providers to become more skilled in sensitively managing the psychiatric comorbidities of patients. Further, Foust et al [29] shared how providers lacked preparation and attention to caregiver needs during transitions from hospital to home.

There is a broad base of literature supporting the need to effectively communicate and coordinate care across sectors. Davis et al [30] found that a lack of communication between members of the care team, as well as across settings, led to poorly executed transitions and negative patient and provider experiences. They recommended clarifying the accountability of team members, standardizing the transition process through multidisciplinary hospital rounds, and training additional medical staff. Our findings supported this and suggested that unregulated care providers and peer support workers be included as members of the care team. Personal Support Workers are unregulated care providers who provide the majority of home and community care in the study context, but are typically undervalued, precariously employed, and overburdened with care responsibility with few (if any) linkages to a broader team of care providers. In terms of peer support workers, there is strong evidence in the mental health literature on the value of this role, particularly, the authenticity of linking two people with a shared experience and associated good outcomes [31].

Finally, the burden and shock of a care transition can be eased through an interim care option. Participants described “temporary” assisted living as a way to ease a difficult transition, or on the other hand, gradually acclimatizing someone accustomed to a higher level of service such as facility based long-term care. This type of transitional setting is akin to the Australian Transition Care Program [32] which offers housing with supports as an “in-between” and realistic “life at home” set-up following a hospital stay and prior to returning home. Support for ADLs and IADLs after people have returned home is important to ease the transition, and additional work to identify, examine and implement innovative models to support patients and their families post discharge is required.

## The Barriers and Workarounds

Importantly, meeting the needs of people with complex care needs requires careful consideration of policy levers and organizational arrangements. Care provider’s work within structures guided by rules, policies, accountability and reporting requirements that effectively shapes and often limits what’s possible in their day to day interactions with patients, other providers, and families. Moving beyond these boundaries or “working the system” becomes necessary in order to meet the unique and fluctuating needs of people with complex care needs, but may be coupled with feelings of dissatisfaction and moral distress [33].

International health systems are strongly advocating for the integration of health and social care, particularly for sub-groups of patients who stand to benefit the most, including those with multimorbidity and complex care needs. This literature highlights the enablers and disablers of such integrated approaches. A recent paper by Maruthappu et al [34] outlined enablers to integrated care including infrastructure for information technology, clinical leadership, the involvement of primary care, a culture shift, accountability and governance, which spans over the full continuum of services as opposed to separate providers and sectors. Financial incentives such as pooled funding and appropriate evaluation and metrics, which support the integration of services, have also been recommended and align with our study findings.

In this paper, we shed light on important nuances that need to be considered when successfully engaging with patients and families in day-to-day care. We highlight the potential utility of formalizing the often-overlooked roles of peers and Personal Support Workers, who play critical roles in meeting both the health and social needs of people, but are often devalued or restricted in their role on care teams. We outline concrete examples of perverse incentives that limit the extent to which providers can deliver on what patients and families need. We recommend that reforming models of care delivery towards ‘integrated’ systems requires the consideration of the complex interactions that care providers have with structural barriers, and the means they use to mitigate them. In this paper, people on the front lines have identified some key opportunities for future work and focus for the care and support of people with complex care needs, and our findings serve as a framework in this endeavor.

## Limitations

While several participants across various health and social care organizations were contacted for participation several did not respond after two attempts. The researchers cast a fairly wide net in anticipation of a high non-response rate given the typically busy schedules of these types of providers. Despite a high non-response rate, the sample garnered perspectives from 24 health and social care providers with a wealth of experience working with complex patients populations. The providers were all from one major urban area and served culturally diverse clients of various ages. Findings, therefore, may be limited to similar geographic settings and client profiles. Despite this, the broad categories are likely applicable beyond the context of the study and will resonate with a range of providers who are seeking to integrate care for people with complex care needs and their families.

## Conclusions

Meeting the needs of people with complex care needs requires authentic and consistent relationships with providers and families and ongoing communication. Attention to non-medical factors including culture, personal goals and expectations, can also provide insight into care preferences and levels of engagement. Teams that recognize and support less formalized roles of families, personal and peer support workers, are critical to the delivery of supports to this population. This type of care delivery model, along with the mobilization of needed health and social care supports can be more effectively realized with the appropriate training, levers and incentives, in place at the organization and policy levels. Perhaps the greatest challenge is the persistent orientation of health systems toward acute and episodic care; equipped to react quickly to problems after they arise, and then move onto the next case. We offer some essential building blocks to address the health and social care needs of complex patient populations, but it will be much easier to put these components into practice within a system that is oriented to proactively supporting health and well-being.
